# Small Intestinal Bacterial Overgrowth in Metabolic Dysfunction-Associated Steatotic Liver Disease: Prevalence, Subtypes, and Risk Factors Across Disease Spectrum and Comorbidity Profiles

**DOI:** 10.3390/biomedicines14051042

**Published:** 2026-05-03

**Authors:** Yangjie Li, Huiping He, Limin Chen, Jing Chen, Man Gu, Yueyan Hu, Lirong Guo, Siheng Long, Jiaying Hu, Zhukun Zhou, Yao Xiao, Zihan Wu, Hongju Yang

**Affiliations:** Geriatric Medical Center, Division of Geriatric Gastroenterology, The First Affiliated Hospital of Kunming Medical University, Kunming 650032, China; liyangjie1020@163.com (Y.L.); hehuiping239@163.com (H.H.); clm1387711@163.com (L.C.); panda20230325@163.com (J.C.); guman@kmmu.edu.cn (M.G.); huyueyan78@sina.com (Y.H.); glirong111@163.com (L.G.); longsiheng@163.com (S.L.); 15928168056@163.com (J.H.); zhouzhukun2001@outlook.com (Z.Z.); xyxm1412@163.com (Y.X.); wuzihan19971207@163.com (Z.W.)

**Keywords:** metabolic dysfunction-associated steatotic liver disease, small intestinal bacterial overgrowth, liver cirrhosis, intestinal methanogen overgrowth, risk factors

## Abstract

**Background**: Small intestinal bacterial overgrowth (SIBO) has been implicated in the pathogenesis of MASLD; however, large-scale clinical data characterizing prevalence patterns, phenotypic subtypes, and disease-specific associations remain limited. **Methods**: This cross-sectional study enrolled 2549 MASLD patients with gastrointestinal symptoms undergoing lactulose methane–hydrogen breath testing and transient elastography. Univariate and multivariable analysis identified independent risk factors for SIBO. We also explore the distribution of SIBO subtypes and their associations with comorbidity profiles across the MASLD spectrum. **Results**: The overall prevalence of SIBO was 66.3%, escalating from 65.9% in MASL to 72.8% in at-risk MASH and 78.9% in cirrhosis, alongside a notable enrichment of the intestinal methanogen overgrowth (IMO) phenotype. Multivariable analysis identified advanced fibrosis (stage F4; OR = 1.75, 95% CI: 1.03–2.96), gastroesophageal reflux disease (GERD; OR = 1.66, 95% CI: 1.22–2.28), and coronary artery disease (CAD; OR = 1.80, 95% CI: 1.06–3.06) as independent predictors of SIBO. Additionally, elevated ALT (OR = 1.01, 95% CI: 1.01–1.13) showed a modest association with SIBO. Subtype analysis revealed that IMO was associated with GERD, alcohol consumption, CAD, and obesity, while a history of cholecystectomy and elevated triglycerides were linked to early-phase hydrogen peaks. **Conclusions**: SIBO is highly prevalent among patients with MASLD, with its prevalence and phenotypic subtype distribution being closely associated with disease severity. The identification of fibrosis-specific risk factors and subtype–clinical associations suggest consideration of SIBO assessment in advanced MASLD, particularly in patients with cardiometabolic or gastrointestinal comorbidities.

## 1. Introduction

Metabolic dysfunction-associated steatotic liver disease (MASLD), previously termed non-alcoholic fatty liver disease, is a chronic liver disease that is closely associated with metabolic dysfunction. The prevalence of MASLD is rising, making it the most common chronic liver disease worldwide and imposing a heavy burden on healthcare systems [1]. MASLD emphasizes the close relationship between the disease and metabolic syndrome, indicating that MASLD is not merely hepatic fat deposition, but rather a spectrum of systemic diseases with insulin resistance at its core, involving genetic–epigenetic factors, metabolic disorders, immune imbalance, and environmental factors, with hepatic manifestations. MASLD encompasses a spectrum of diseases, ranging from metabolic-associated steatotic liver (MASL, formerly known as simple steatosis) and metabolic-associated steatohepatitis (MASH) to progression to hepatic fibrosis and cirrhosis [2,3].

Over the past decade, the intestinal microecology has been regarded as a “metabolic organ,” engaging in continuous bidirectional communication with the liver through the gut–liver axis. Intestinal microbiota influences the hepatic lipid metabolism by regulating host fatty acid synthesis and cholesterol metabolism, while the liver reciprocally regulates intestinal microecological balance through bile acids [4]. Accumulating evidence demonstrates that MASLD patients commonly exhibit characteristic gut dysbiosis, marked by decreased microbial diversity, a depletion of beneficial bacteria, and an overgrowth of pathogenic taxa. These changes subsequently activate Toll-like receptor (TLR) signaling pathways in hepatic Kupffer cells, triggering a systemic inflammatory state that is characteristic of metabolic syndrome and accelerating hepatic fat deposition and fibrogenesis [5,6,7]. The bidirectional link between the gut microbiota and MASLD has become a research hotspot that is of considerable interest [8]. However, previous studies have primarily focused on fecal microbiota, with insufficient attention to small intestinal microecology.

Under physiological conditions, gastric acid, bile acids, pancreatic enzymes, and intestinal motility collectively maintain a near-sterile environment in the small intestine. Gastrointestinal dysfunction, obesity, high-fat diets, diabetes mellitus, and various other factors can weaken these defense mechanisms, leading to SIBO. SIBO causes alterations in the gut microbiome and increased intestinal permeability, enabling microbial metabolites and pathogenic factors to enter the liver [9]. Clinical studies have shown that MASLD patients with SIBO exhibit significantly elevated serum endotoxin levels; lipopolysaccharide (LPS) activates hepatic nuclear factor-κB (NF-κB) and Toll-like receptor 4 (TLR-4) pathways, driving inflammation and fibrosis [10]. Furthermore, SIBO can induce disorders in bile acid metabolism, abnormal regulation of short-chain fatty acids (SCFAs), and impaired choline absorption, thereby affecting fat uptake and exacerbating intrahepatic fat accumulation [11]. SIBO may influence the progression of MASLD through mechanisms including disruption of gut–liver axis homeostasis, increased endotoxin translocation, activation of hepatic inflammation, and exacerbation of metabolic disorders [12]. Multiple cross-sectional studies have confirmed that the prevalence of SIBO in MASLD patients is significantly higher than in healthy controls [13,14]. A systematic review and meta-analysis comprising 14 studies demonstrated a clear association between SIBO and MASLD, with the probability of SIBO positivity increasing with the severity of MASLD pathological changes [15].

As established, an epidemiological association and pathophysiological link between SIBO and MASLD have been reported. However, current data regarding the specific prevalence of SIBO in MASLD patients remain inconsistent, and the study sample sizes are relatively small. The present study utilizes a large-sample, real-world clinical research approach to comprehensively analyze the relationship between MASLD and SIBO, systematically elucidating the following key aspects: the prevalence of SIBO in MASLD patients; assessment of SIBO occurrence and SIBO subtype distribution differences across different disease progression stages in MASLD patients (MASL, MASH, and cirrhosis); independent risk factors for SIBO development in MASLD patients; and SIBO classification. This study aims to provide novel perspectives and evidence for precise stratification, clinical diagnosis, intervention, and treatment of MASLD patients with concurrent SIBO. This study sought to determine the prevalence and distribution of SIBO subtypes across the MASLD spectrum and elucidate their associations with the biochemical markers of liver injury, metabolic profiles, and extrahepatic comorbidities.

## 2. Methods

### 2.1. Study Population

**Figure 1:**
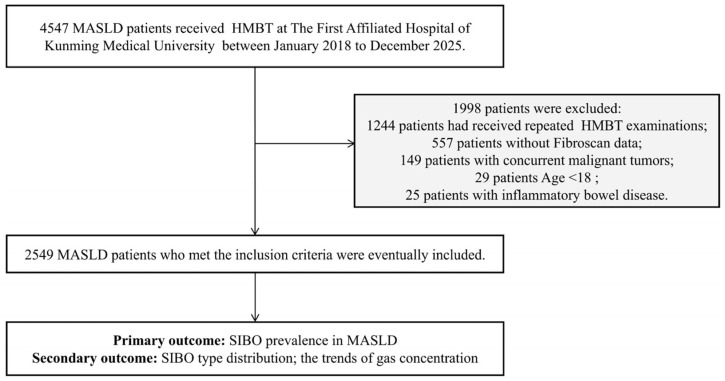
**Study flow diagram and patient selection criteria.** This diagram illustrates the detailed inclusion and exclusion process for this retrospective cross-sectional cohort.

MASLD patients who underwent transient elastography and a hydrogen–methane breath test (HMBT) were included from January 2018 to December 2025 at the Department of Geriatric Gastroenterology and Gastroenterology in the First Affiliated Hospital of Kunming Medical University. The diagnosis of MASLD was made in accordance with the 2024 EASL-EASD-EASO Clinical Practice Guidelines [16], requiring the presence of metabolic abnormalities and the absence of other etiologies for steatosis. This guideline places greater emphasis on the metabolic origin of the disease and does not exclude the coexistence of other liver diseases or moderate alcohol intake. However, we strictly excluded patients with documented concurrent liver diseases to avoid confounding etiologies. Based on the results of baseline imaging, and/or biochemical, and/or histological assessments, all patients were categorized into three subgroups: MASL, at-risk MASH, and metabolic-associated cirrhosis. MASL was defined as hepatic steatosis involving ≥5% of hepatic tissue confirmed by imaging or pathological examination of liver biopsy; at-risk MASH was defined as a Non-Alcoholic Fatty Liver Disease Activity Score (NAS) ≥ 4 with a fibrosis stage ≥ 2; and metabolic-associated steatotic liver cirrhosis was defined as MASLD progression to cirrhosis. The inclusion criteria for this retrospective study were as follows: age ≥ 18 years and MASLD patients who had undergone hydrogen–methane breath testing and Fibroscan examination with complete medical records. The exclusion criteria included: a history of major gastrointestinal or other abdominal surgery; comorbidity with inflammatory bowel disease, intestinal obstruction, short bowel syndrome, or other diseases that may severely impair gastrointestinal function; and pregnant or lactating women. Below is the study flow diagram and patient selection criteria (Figure 1).

### 2.2. Data Acquisition

The demographic and clinical characteristics were extracted from the electronic medical record system. These included personal information (age, sex, and body mass index [BMI]), lifestyle factors (smoking and alcohol consumption), past medical history, and comorbidities (e.g., Helicobacter pylori infection, gastric/intestinal polyps, chronic atrophic gastritis [CAG], coronary artery disease [CAD], hyperlipidemia, hypertension, diabetes, gastroesophageal reflux disease [GERD], and a history of appendectomy or cholecystectomy). The laboratory data included liver function indices (alanine aminotransferase [ALT], aspartate aminotransferase [AST], and total bilirubin [TBIL]) and metabolic profiles (fasting blood glucose [FBG], total cholesterol [TC], fasting triglycerides [TG], high-density lipoprotein [HDL], and low-density lipoprotein [LDL]). Because liver biopsies were performed only in a small subset of patients with specific clinical indications, we calculated the FibroScan-AST (FAST) score and Fibrosis-4 (FIB-4) index based on relevant clinical parameters to assist in evaluating the liver disease severity.

All patients underwent transient elastography based on the FibroScan™ device (Echosens, Paris, France) to assess the degree of hepatic steatosis and liver fibrosis, which were quantified by the controlled attenuation parameter (CAP, dB/m) and liver stiffness measurement (LSM), respectively. Based on LSM (E value), MASLD liver fibrosis was graded as normal/mild scarring (F0–F1: <8.2 kPa), moderate scarring (F2: 8.2–9.6 kPa), severe scarring (F3: 9.7–13.5 kPa), and cirrhosis (F4:  ≥13.6 kPa) [17]. Meanwhile, hepatic steatosis grading relies on CAP. The CAP value of  ≥238 dB/m indicated hepatic steatosis or MASLD. Based on CAP, hepatic steatosis or MASLD was graded as mild (S1: 238–259 dB/m), moderate (S2: 260–292 dB/m), and severe (S3:  ≥293 dB/m). These values were provided by the FibroScan™ manufacturer (Echosens, Paris, France).

### 2.3. HMBT

MASLD patients with significant gastrointestinal symptoms (bloating, abdominal pain, early satiety, altered bowel habits, or excessive flatulence) underwent HMBT. The diagnosis of SIBO traditionally relies on a bacterial count of ≥10^5^ CFU/mL in cultured small intestinal fluid aspirates. However, due to the invasive nature of small intestinal fluid culture, its clinical application is limited [18]. The HMBT is widely recognized as a non-invasive method for diagnosing SIBO [19]. Therefore, all patients underwent the HMBT, following the standard test procedures.

All subjects were instructed to adhere to a low-fermentable diet for 12 h before the test and to avoid high-fiber foods, dairy products, carbonated drinks, and alcohol. Antibiotics, probiotics, laxatives, prokinetic agents, and proton pump inhibitors were discontinued for at least 2 weeks to eliminate intestinal flora interference. They were required to rinse their mouths thoroughly and brush their teeth to maintain oral hygiene before the test. After 15 min of quiet sitting rest to stabilize their respiratory and metabolic status, the participant’s baseline end-expiratory breath sample was collected using a sterile disposable gas collection tube. The participants then orally ingested 15 mL of lactulose dissolved in 100 mL of warm water. After lactulose intake, breath samples were collected at 20 min intervals for a total test duration of 120 min, and the gas concentrations in the exhaled breath samples were expressed in parts per million (ppm). All breath samples were analyzed by a calibrated automatic gas chromatograph to determine the hydrogen (H_2_) and methane (CH_4_) concentrations and time–concentration curves were plotted. All breath test results were independently assessed by two trained technicians. Any disagreements were resolved by a discussion. This dual-review process guaranteed the reliability and consistency of the interpretations.

### 2.4. Outcomes

The primary outcome was the SIBO prevalence in MASLD patients and the definition of SIBO was based on the 2020 North American Consensus [20]. The diagnostic criteria for SIBO are as follows: (i) H_2_ positive—an increase of ≥20 ppm in hydrogen concentration from the baseline within 90 min; (ii) CH_4_ positive—an increase of ≥10 ppm in methane concentration from the baseline within 90 min; (iii) since methanogens consume hydrogen to produce CH_4_, to improve the accuracy of breath tests, manufacturers have established a combined diagnostic criterion: H_2_ + CH_4_ > 15 ppm within 30 min, or H_2_ + CH_4_—baseline concentration > 15 ppm within 2 h. Meeting any one of these three conditions is sufficient to diagnose positive SIBO. If any of the above criteria are met, the result is considered positive; otherwise, it is negative. Due to laboratory reporting constraints, the rise in baseline hydrogen was evaluated at the 100 min mark, rather than the standard 90 min cutoff recommended by the guidelines.

The secondary outcomes included: investigating the prevalence and types of SIBO across different stages of MASLD (including MASL, at-risk MASH, and MASH-associated cirrhosis), as well as comparing the trends of CH_4_ and H_2_ gas levels over 120 min and the time to peak gas concentration in MASLD patients. The diagnosis of SIBO using HMBT can be categorized into H_2_-producing and CH_4_-producing types. The H_2_-producing type was defined as H_2_ positive; CH_4_-producing types were defined as CH4 positive. If a patient was positive for both hydrogen and methane, it was defined as mixed-type SIBO. Since methanogens are not bacteria but archaea, and their overgrowth is not limited to the small intestine but can also extend to the large intestine, the American Gastroenterological Association guidelines propose replacing the term “CH_4_-producing SIBO” with “intestinal methanogen overgrowth” (IMO). The time to peak gas concentration was categorized as an early peak (≤60 min) or a late peak (>60 min), based on a 60 min cutoff.

### 2.5. Statistical Analysis

All analyses were conducted using R software (version 4.5.2). Continuous variables were summarized as mean ± standard deviation (SD) for normally distributed data or median (interquartile range [IQR]) for non-normally distributed data. Categorical variables were expressed as frequencies (n) and percentages (%). Group comparisons for continuous variables utilized independent sample *t*-tests or Mann–Whitney U tests (for non-normal distributions), while categorical variables were compared using χ^2^ or Fisher’s exact tests, as appropriate. Multivariable logistic regression models were constructed to identify the independent predictors of SIBO positivity. Candidate variables for multivariable analysis were selected based on univariate associations (*p* < 0.05) and clinical relevance. Results are reported as odds ratios (OR) with 95% confidence intervals (CI) and two-sided *p* values. *p*-value < 0.05 was considered statistically significant.

## 3. Results

### 3.1. Patients’ Characteristics

A total of 2549 MASLD patients who underwent HMBT and transient elastography assessment were enrolled, based on the inclusion and exclusion criteria (Figure 1). The baseline characteristics are summarized in Table 1. The cohort comprised 2040 (80.0%) patients with MASL, 367 (14.4%) with at-risk MASH, and 142 (5.6%) with cirrhosis. The patients with cirrhosis were significantly older than those with MASL and at-risk MASH groups (*p* = 0.003), whereas no significant differences were observed among groups regarding gender distribution, smoking status, or drinking (*p* > 0.05).

Liver disease severity indices exhibited progressive deterioration across the MASLD spectrum. The CAP values peaked in at-risk MASH compared to the cirrhosis and MASL groups (*p* < 0.001). Non-invasive fibrosis scores, including LSM (E scores), FAST scores, and FIB-4 scores, progressively increased from MASL to cirrhosis (*p* < 0.001). ALT was significantly elevated in at-risk MASH and cirrhosis compared to MASL (*p* < 0.001), while AST increased progressively across all groups (*p* < 0.001). TBIL levels reached their highest point in the cirrhosis group (*p* < 0.001).

Metabolic parameters also diverged by disease stage. BMI and fasting glucose were higher in at-risk MASH and cirrhosis than in MASL (*p* < 0.001). TG were highest in at-risk MASH and lowest in cirrhosis, whereas TC and LDL-C were reduced in cirrhosis, relative to earlier stages (*p* < 0.001). The prevalence of CAD (*p* < 0.001), diabetes (*p* < 0.001), and GERD (*p* = 0.034) increased significantly as the disease progressed from MASL to cirrhosis. In contrast, no significant differences were observed for Helicobacter pylori infection, gastric/intestinal polyps, CAG, hyperlipidemia, hypertension, or history of appendectomy/cholecystectomy (*p* > 0.001).

### 3.2. SIBO Prevalence Increases with MASLD Severity and Associates with Advanced Fibrosis

**Figure 2:**
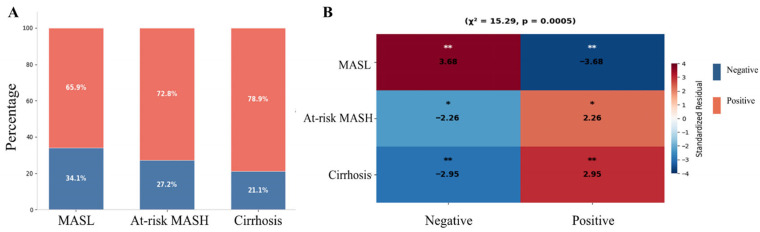
**SIBO positivity across MASLD spectrum.** (**A**) Stacked bar chart showing the positive rate of SIBO across three MASLD stages. (**B**) Heatmap of standardized residuals from chi-square test analysis (χ^2^ = 15.29, *p* = 0.0005) examining association between SIBO status and disease stage. Standardized residuals > |2| indicate significant deviation from expected frequencies. * *p* < 0.05, ** *p* < 0.01.

Among 2549 patients with MASLD, the prevalence of SIBO was 67.63% (1724/2549). The positive rate for SIBO increased progressively from MASL at 65.9%, to at-risk MASH at 72.8%, and further to liver cirrhosis at 78.9%; correspondingly, the SIBO-negative rate showed a significant decline (Figure 2A). A heatmap of standardized residuals indicated that MASL was significantly negatively associated with SIBO, whereas at-risk MASH and liver cirrhosis demonstrated significant positive associations (Figure 2B).

To investigate the risk factors for SIBO prevalence in MASLD patients, all patients were further categorized into SIBO positive and negative groups. MASLD patients with SIBO positivity were older (*p* = 0.033) and exhibited a higher BMI (*p* = 0.003). Additionally, patients with SIBO positivity showed higher FAST scores (*p* < 0.001), FIB-4 scores (*p* = 0.016), ALT levels (*p* = 0.043), and AST levels (*p* = 0.021). Furthermore, the prevalence of drinking was higher among SIBO positive patients (*p* = 0.013), as was the prevalence of intestinal polyps (*p* = 0.010), CAD (*p* < 0.001), diabetes (*p* = 0.020), and GERD (*p* < 0.001). The distribution of the F stages differed significantly between the groups (*p* = 0.010) (Table 2).

**Figure 3:**
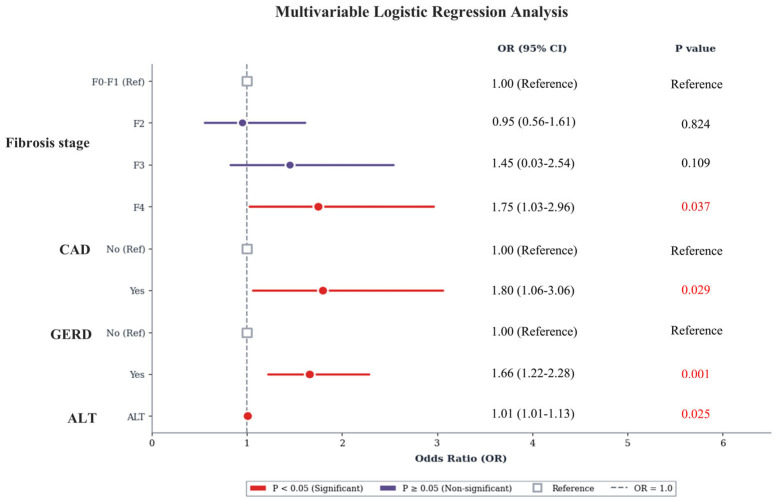
**Multivariable logistic regression analysis for SIBO positivity**.

Multivariable logistic regression analysis identified independent risk factors for SIBO positivity (Figure 3). Compared with the F0–F1 stage, the F4 stage was significantly associated with increased odds of SIBO (OR 1.75, 95% CI 1.03–2.96, *p* = 0.037), whereas the F2 and F3 stages showed no significant association (*p* > 0.05). Additionally, the presence of CAD (OR 1.80, 95% CI 1.06–3.06, *p* = 0.029) and GERD (OR 1.66, 95% CI 1.22–2.28, *p* = 0.001) emerged as independent predictors. Elevated ALT levels also demonstrated a modest but statistically significant association with SIBO (OR 1.01, 95% CI 1.01–1.13, *p* = 0.025).

### 3.3. SIBO Subtypes in MASLD Patients

**Figure 4:**
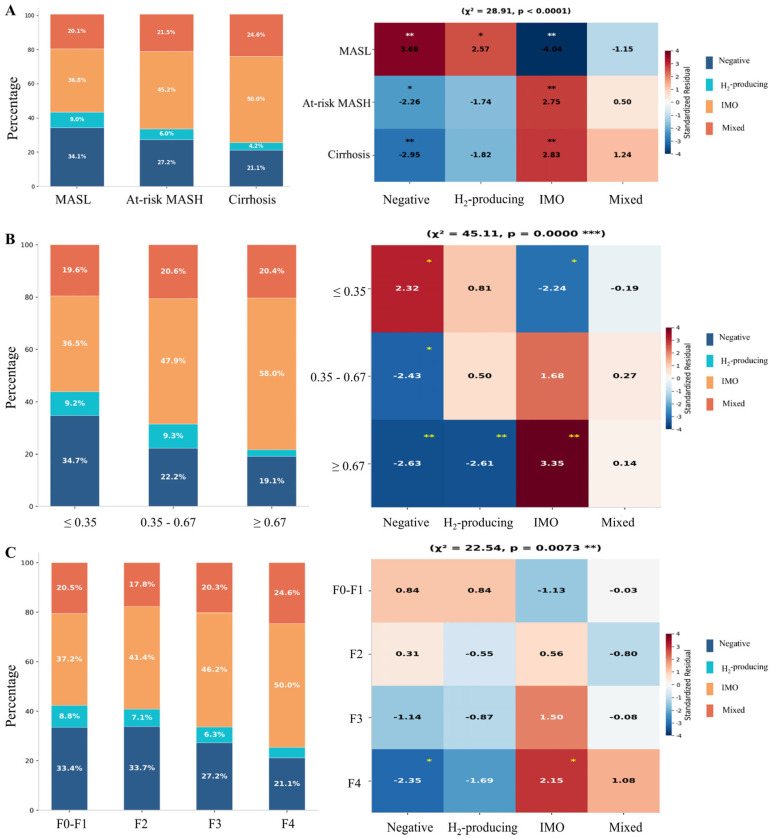
**SIBO subtype across MASLD spectrum.** Stacked bar charts (left) and standardized residual heatmaps (right) showing SIBO subtype distribution across (**A**) MASLD stages (χ^2^ = 28.91, *p* < 0.0001), (**B**) FAST score categories (≤0.35, 0.35–0.67, ≥0.67; χ^2^ = 45.11, *p* < 0.0001), and (**C**) fibrosis stages (F0–F1, F2, F3, F4). Standardized residuals > |2| indicate significant deviation from expected frequencies. * *p* < 0.05, ** *p* < 0.01, and *** *p* < 0.001.

A total of 825 (32.37%) MASLD patients were classified as negative, 212 (8.32%) as H_2_-producing, 988 (38.76%) as IMO, and 524 (20.56%) as mixed. The SIBO subtype distribution varied significantly across the MASLD spectrum (Figure 3). From MASL to cirrhosis, IMO and mixed-type SIBO increased from 36.8% and 20.1% to 50.0% and 24.6%, respectively, while negative cases decreased from 34.1% to 21.1% (χ^2^ = 28.91, *p* < 0.0001, Figure 4A). With stratification by non-invasive fibrosis risk categories’ FAST scores ≤ 0.35, 0.35–0.67, and ≥0.67), a significant shift toward IMO dominance was observed in the high FAST group (≥0.67), and IMO accounted for 58.0% of cases in the high FAST group (≥0.67) compared to 36.5% in the low FAST group (≤0.35). Standardized residual analysis confirmed significant enrichment of IMO and depletion of SIBO-negative and H_2_-producing cases in the high-risk group (Figure 4B). Consistently, the F4 stage showed IMO enrichment (50.0% vs. 37.2% at F0–F1, χ^2^ = 22.54, *p* = 0.0073), confirming that IMO accumulation parallels MASLD progression (Figure 4C). Collectively, these findings demonstrate a progressive enrichment of IMO and mixed-type SIBO during MASLD progression and hepatic fibrosis worsening.

Meanwhile, SIBO subtypes exhibited distinct distribution patterns across various clinical and demographic parameters (Figure S1). Gender-stratified analysis demonstrated distinct phenotypic patterns: female patients exhibited higher rates of H_2_-producing SIBO compared to males, whereas male patients showed a predominance of IMO. Regarding metabolic and lifestyle factors, alcohol consumption was associated with significant IMO enrichment. Obese patients (BMI > 28 kg/m^2^) and patients with CAD showed significant IMO enrichment and significant reduction in SIBO-negative prevalence.

For gastrointestinal diseases, MASLD patients with GERD demonstrated the strongest association with SIBO phenotypes. GERD patients exhibited marked IMO enrichment and significant depletion of SIBO-negative cases. Similarly, patients with intestinal polyps exhibited a contrasting pattern characterized by significant IMO enrichment and reduced H_2_-type SIBOa (Figure S2). No significant differences were observed among groups for variables including the S stage, TBIL, glucose, LDL, HDL, smoking, Hp infection, gastric polyps, chronic gastritis, hyperlipidemia, hypertension, diabetes, and appendectomy (*p* > 0.05).

### 3.4. Predictors of Early Bacterial Overgrowth in MASLD Patients

**Figure 5:**
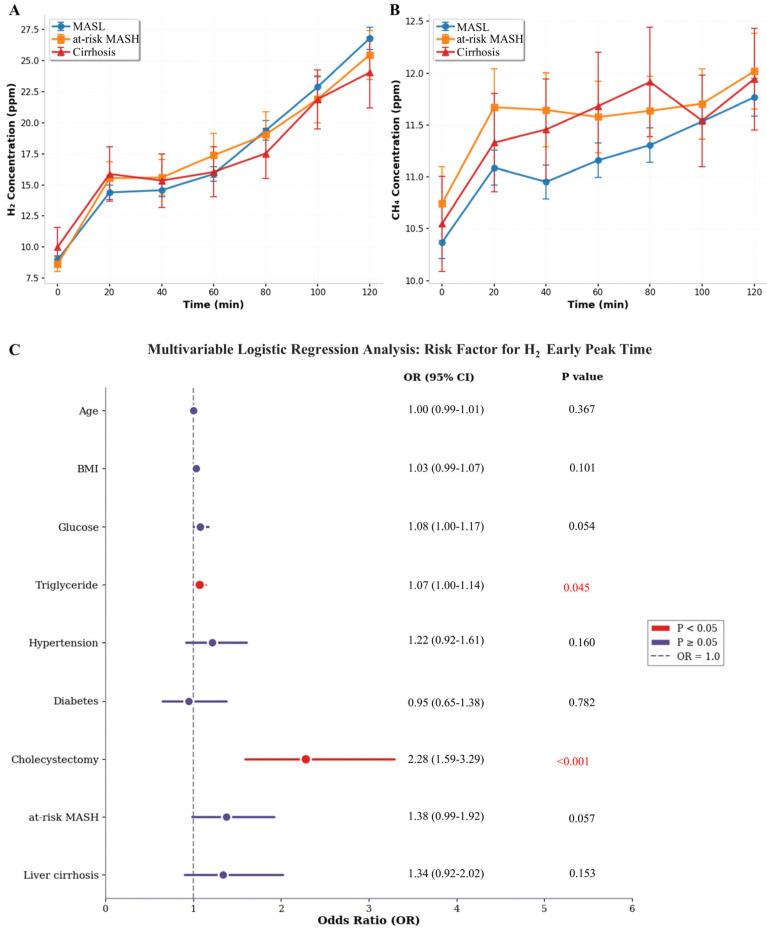
**Hydrogen and methane breath test kinetics and risk factors for early hydrogen peak across MASLD spectrum.** Time-course curves of breath H_2_ (**A**) and CH_4_ (**B**) concentrations following lactulose ingestion (0–120 min) in MASLD patients. (**C**) Forest plot of multivariable logistic regression analysis showing independent predictors of early H_2_ peak time (defined as peak concentration occurring within 60 min).

The HMBT revealed distinct fermentation patterns across the MASLD spectrum. The H_2_ concentration curves demonstrated that at-risk MASH and cirrhosis patients exhibited higher early-phase H_2_ levels (20–60 min), whereas the MASL group showed a progressive increase throughout the 120 min observation period, ultimately achieving the highest concentrations at the terminal time point (Figure 5A). Conversely, CH_4_ kinetics indicated that cirrhosis patients maintained persistently elevated baseline and fluctuating methane levels compared to MASL and at-risk MASH groups, suggesting a higher prevalence of IMO in advanced liver disease (Figure 5B).

The time to peak gas concentration was categorized as an early peak (≤60 min) or a late peak (>60 min), based on a 60 min cutoff. Within the 60 min timeframe, the characteristics of the CH_4_ peak showed that 57.3% were classified as early peaks and 42.7% as late peaks, with no significant association found with disease severity or biochemical markers. Regarding H_2_, the peak timing indicated that 58.8% were early peaks, while 41.2% were late peaks. Univariate analysis revealed that age, BMI, hypertension, diabetes, fasting glucose, TG, cholecystectomy, at-risk MASH, and cirrhosis were associated with early H_2_ peak timing. Multivariable logistic regression analysis identified specific clinical and metabolic factors associated with early H_2_ peak time, which was indicative of proximal SIBO (Figure 4C). History of cholecystectomy emerged as the strongest independent risk factor (OR 2.28, 95% CI 1.59–3.29, *p* < 0.001). Additionally, an elevated TG level demonstrated a modest but significant association with an early H_2_ peak (OR 1.07, 95% CI 1.00–1.14, *p* = 0.045).

## 4. Discussion

SIBO is characterized by pathological colonization of the small intestine and has been implicated in gastrointestinal dysfunction, pancreatitis, liver disease, metabolic disorders, and impaired lipid and nutrient absorption [21,22,23]. Accumulating evidence demonstrates correlations among SIBO, gut dysbiosis, bacterial translocation, and hepatic steatosis in MASLD; however, the existing evidence derives predominantly from small-scale studies. The present large-scale cross-sectional study of 2549 patients reveals an escalating prevalence of SIBO with advancing MASLD severity. This is accompanied by a marked phenotypic shift across the disease spectrum, characterized by the progressive depletion of SIBO-negative cases and a concurrent enrichment of both IMO and mixed-type SIBO in advanced MASLD, particularly in cirrhosis. Furthermore, subtype-specific analysis revealed distinct clinical correlates for IMO, demonstrating significant associations with GERD, alcohol consumption, and obesity.

Although the precise pathophysiological relationships between SIBO and MASLD, including disease staging and progression, remain incompletely elucidated, multiple studies have demonstrated that patients with MASLD exhibit significantly higher SIBO prevalence than healthy controls, with a pooled odds ratio of 3.82 (95% CI: 1.93–7.59) for concurrent SIBO [24]. Moreover, SIBO prevalence appears to escalate with increasing liver disease severity. Compared with patients having simple steatosis, those with nonalcoholic steatohepatitis [25], significant hepatic fibrosis [26], and NASH-related cirrhosis [13] demonstrate incrementally higher SIBO rates. Consistently, a study of obese patients with MASLD revealed that those with MASH and concomitant hepatic fibrosis exhibited significantly higher SIBO prevalence (65.2%) than those without fibrosis (33.3%) [14]. Shi et al. further reported that SIBO prevalence increased correspondingly with steatosis severity as measured by CAP, ranging from 32.6% in mild to 88% in severe steatosis [27], suggesting a quantitative association between bacterial overgrowth and disease progression, particularly fibrogenesis. Our data corroborate these observations, demonstrating a high overall SIBO prevalence (66.3%) that increased progressively from MASL to at-risk MASH and in cirrhosis. Notably, the SIBO positivity rate in our cohort is higher than that reported in the previous literature. As a tertiary care center, our cohort consisted of symptomatic patients (bloating, abdominal pain, and altered bowel habits) who were referred for clinically indicated HMBT. This enrichment of symptomatic individuals likely overestimates the SIBO prevalence compared to the asymptomatic MASLD population, which limits the generalizability of our findings. Secondly, the use of lactulose as a substrate generally yields higher SIBO detection rates than glucose-based protocols [28,29]. Meanwhile, extending the interpretation threshold to 100 min may influence the hydrogen-positive rate compared to the standard 90 min cutoff.

Patients with MASLD and concomitant SIBO exhibited significantly higher FAST scores, FIB-4 indices, and AST levels. Notably, Fibrosis stage F4 and elevated ALT were regarded as independent predictors of SIBO positivity in our multivariable model. Several mechanisms may underlie this observed gradient. First, advancing liver disease impairs bile acid synthesis and secretion, thereby diminishing the antimicrobial properties of bile salts and facilitating proximal small bowel colonization. Second, portal hypertension in cirrhosis induces intestinal mucosal edema and dysmotility, creating a permissive environment for bacterial proliferation. Third, SIBO-mediated disruption of intestinal barrier integrity enhances bacterial translocation and endotoxemia, subsequently activating hepatic TLR and NF-κB pathways, affecting hepatic inflammation and fibrogenesis [10,30]. This establishes a vicious cycle wherein advanced liver disease promotes SIBO, which in turn exacerbates hepatic injury. While SIBO may represent a potentially modifiable risk factor, the retrospective, cross-sectional design of this study means that our findings establish only an association between the two conditions; they cannot be used to infer that SIBO directly drives the progression of MASLD fibrosis. Furthermore, although ALT was independently associated with the presence of SIBO, the effect size was small. This suggests that elevated ALT more likely reflects underlying hepatic inflammation, rather than serving as a strong, independent clinical predictor. Overall, these results indicate that the early identification of SIBO in patients with advanced MASLD could have meaningful implications for disease management.

Furthermore, patients with MASLD and comorbid GERD exhibited significantly higher SIBO positivity rates, with GERD identified as an independent risk factor. Previous studies have demonstrated significantly elevated SIBO prevalence in patients with GERD compared with healthy populations [31], with GERD representing an independent risk factor for SIBO positivity. Notably, our results indicated that the association between GERD and IMO appears stronger than that with H_2_-dominant SIBO. In agreement with this finding, a retrospective study of 394 patients found GERD to be more prevalent among SIBO-positive individuals, with this association being significantly stronger in those with positive methane breath tests than in those with positive hydrogen breath tests [32]. Additionally, our previous work demonstrated that patients with refractory GERD exhibited significantly higher methane breath values than those with non-refractory disease, with elevated methane levels predicting treatment resistance [33]. The robust GERD-IMO association likely reflects alterations in the gastric pH and proximal gut microbiome ecology that favor methanogenic archaea colonization. This GERD-IMO axis may represent a distinct pathophysiological subset requiring targeted microbiota-directed intervention.

MASLD seldom occurs in isolation but rather clusters with metabolic perturbations within an interconnected pathophysiological network encompassing insulin resistance, dyslipidemia, obesity, metabolic syndrome, and CAD [34,35]. Studies have shown that SIBO-positive T2DM patients exhibit markedly lower early-phase and total insulin secretion indices, as well as reduced insulin sensitivity [36]. Consistent with this, our study demonstrated progressive deterioration in metabolic health across the disease spectrum, evidenced by significantly higher BMI and fasting glucose levels in patients with at-risk MASH and cirrhosis compared with those with MASL, alongside increased prevalence of CAD and diabetes. SIBO exacerbates cardiometabolic risk through the promotion of systemic inflammation, impairment of nutrient absorption, and disruption of host metabolic pathways via bacterial metabolites. Conversely, diabetes and its associated hyperglycemic state can induce autonomic neuropathy, leading to significantly reduced gastrointestinal motility and prolonged transit time. Fialho et al. previously reported substantially higher CAD prevalence in SIBO-positive compared with SIBO-negative patients (78.9% versus 38.6%), with a severity-dependent correlation [37]. Demographic analysis revealed that MASLD patients with SIBO were significantly older and exhibited higher BMI, which was consistent with the established SIBO risk factors [38,39,40]. Although SIBO prevalence did not differ significantly between sexes, distinct gender-specific phenotypic patterns emerged: female patients predominantly exhibited H_2_-producing SIBO, whereas male patients showed IMO preponderance. This divergence likely reflects behavioral differences, as male patients demonstrated higher rates of alcohol consumption, which correlated with IMO and mixed-type SIBO positivity. IMO exhibited associations with metabolic comorbidities including obesity and diabetes, though its specificity requires further validation.

The gallbladder plays a critical role in bile acid storage and secretion, which are processes that are essential for lipid digestion and the maintenance of antimicrobial activity within the small intestine. Its removal disrupts the enterohepatic circulation of bile acids, leading to altered gut motility, reduced luminal bile acid concentrations, and subsequent SIBO development. Retrospective and prospective studies have demonstrated significantly elevated SIBO prevalence in post-cholecystectomy patients compared with healthy controls [41]. A retrospective analysis of 1461 patients with SIBO revealed cholecystectomy as being a significant risk factor for hydrogenic SIBO (OR = 1.42; 95% CI 1.06–1.91; *p* = 0.020) but not methanogenic SIBO [42]. Our study identified cholecystectomy history as the strongest independent predictor of an early hydrogen peak, reinforcing the concept that alterations in the bile acid pool following gallbladder removal facilitate proximal small intestinal colonization through the abrogation of bile salt antimicrobial properties. The pathogenesis of post-cholecystectomy SIBO is multifactorial, involving bile acid dysregulation, altered gut microbiota composition, and impaired intestinal motility.

The strengths of this study include the large sample size, comprehensive phenotyping utilizing standardized breath testing and transient elastography, and detailed subtype analysis. However, several limitations warrant acknowledgment. The retrospective, single-center design limits generalizability and precludes causal inference regarding the relationship between SIBO and MASLD progression. Specifically, the cross-sectional nature of our study prevents us from establishing the temporal sequence of SIBO acquisition and MASLD development, nor can we determine whether SIBO represents a cause, consequence, or merely an epiphenomenon of advanced liver disease. Longitudinal cohort studies are warranted to disentangle these complex relationships. Furthermore, our study included MASLD patients who underwent breath testing in a clinical setting and frequently presented with gastrointestinal symptoms. This inherently enriched the study population for SIBO-positive cases, thereby limiting the generalizability of the reported prevalence to the broader asymptomatic MASLD population. Selection bias toward symptomatic patients in a tertiary care center likely overestimates true SIBO prevalence in the general MASLD population. Our findings should be interpreted as being applicable to symptomatic MASLD patients, rather than the entire disease spectrum. Additionally, the interpretation of HMBT is sensitive to intestinal transit time, and the optimal time window remains a subject of ongoing debate [43]. Due to laboratory constraints, we employed a 100 min cutoff for hydrogen positivity—10 min beyond the North American Consensus standard. While consistently applied, this deviation may have captured late colonic fermentation signals, potentially resulting in the modest overestimation of hydrogen-positive SIBO rates. Furthermore, breath testing reflects gas-production phenotypes, rather than direct microbial composition. The lack of culture-based validation or 16S rRNA sequencing limits our ability to characterize specific bacterial taxa or methanogenic archaea species associated with different SIBO subtypes. Future studies integrating metagenomic analysis with breath testing would provide mechanistic insights into the microbial drivers of IMO and hydrogenic SIBO in MASLD. Finally, although we adhered to the standard protocol by discontinuing proton pump inhibitors, antibiotics, probiotics, laxatives, and prokinetics for at least two weeks prior to the breath test, we lacked long-term data on concurrent medications such as metformin, GLP-1 agonists, and statins that affect metabolic parameters. This absence represents a potential unmeasured confounder that may influence SIBO prevalence. Despite these limitations, our large sample size, standardized breath testing protocol, and comprehensive phenotyping using transient elastography provide valuable real-world evidence regarding SIBO-MASLD associations. We have transparently reported these constraints to facilitate an appropriate interpretation of our findings.

## 5. Conclusions

This large-scale cross-sectional study demonstrates that SIBO is highly prevalent in patients with MASLD. Furthermore, its overall prevalence and specific subtype distribution are significantly associated with disease severity and comorbidities: notably, advanced fibrosis, CAD, and GERD. We observed a progressive enrichment of the IMO phenotype alongside MASLD progression. These findings position SIBO not merely as a gastrointestinal curiosity but as a potentially modifiable risk factor in MASLD pathogenesis, underscoring the importance of integrating SIBO assessment into multidisciplinary clinical management of MASLD. Future longitudinal studies are required to disentangle the causal sequence among SIBO acquisition, SIBO subtype evolution, and MASLD development.

## Figures and Tables

**Table 1:** Baseline demographic and laboratory characteristics of MASLD patients.

Variables	MASL	At-Risk MASH	Liver Cirrhosis	*p*
Age	53.53 ± 13.82	52.50 ± 14.14	57.15 ± 13.02	0.003
BMI	24.80 ± 3.18	26.08 ± 3.48	26.12 ± 3.56	<0.001
CAP	291.02 ± 36.43	308.12 ± 39.29	297.24 ± 43.43	<0.001
E score	5.10 (4.30, 6.10)	9.40 (8.50, 10.70)	17.75 (14.80, 25.30)	<0.001
FAST score	0.10 (0.05, 0.20)	0.46 (0.28, 0.59)	0.71 (0.62, 0.81)	<0.001
FIB-4 score	1.24 (0.92, 1.71)	1.47 (0.99, 2.29)	2.68 (1.69, 5.33)	<0.001
ALT	21.15 (14.50, 32.18)	38.70 (23.50, 68.10)	40.60 (25.60, 70.70)	<0.001
AST	21.90 (18.20, 28.30)	34.20 (23.90, 48.80)	44.15 (32.15, 64.25)	<0.001
TBIL	11.90 (8.93, 16.30)	12.75 (9.90, 17.70)	16.45 (11.72, 22.80)	<0.001
FBG	5.44 ± 1.54	5.86 ± 2.22	5.77 ± 1.75	0.002
TC	4.67 ± 1.07	4.62 ± 1.21	4.25 ± 1.44	<0.001
LDL	2.89 ± 1.09	2.82 ± 0.96	2.38 ± 0.98	<0.001
HDL	1.15 ± 0.32	1.09 ± 0.33	1.13 ± 0.46	0.096
TG	1.69 (1.23, 2.38)	1.82 (1.35, 2.54)	1.46 (1.11, 2.21)	0.024
Male	1350 (66.18)	237 (64.58)	94 (66.20)	0.836
Smoke	671 (32.89)	127 (34.60)	48 (33.80)	0.804
Drinking	533 (26.13)	101 (27.52)	42 (29.58)	0.597
Hp	435 (23.64)	76 (23.60)	24 (17.52)	0.259
Gastric polyps	91 (4.46)	10 (2.72)	3 (2.11)	0.144
Intestinal polyps	290 (14.22)	43 (11.72)	16 (11.27)	0.302
CAG	155 (7.60)	22 (5.99)	8 (5.63)	0.411
CAD	123 (6.03)	27 (7.36)	20 (14.08)	<0.001
Hyperlipidemia	349 (17.11)	60 (16.35)	17 (11.97)	0.279
Hypertension	391 (19.17)	77 (20.98)	37 (26.06)	0.114
Diabetes	184 (9.02)	50 (13.62)	38 (26.76)	<0.001
GERD	384 (18.82)	90 (24.52)	31 (21.83)	0.034
Appendectomy	96 (4.71)	14 (3.81)	9 (6.34)	0.473
Cholecystectomy	146 (7.16)	20 (5.45)	16 (11.27)	0.073

Note: Continuous variables are now clearly labeled as either means ± SD for normally distributed data or medians (interquartile range) for non-normally distributed data. Categorical variables are presented as n (%). Abbreviations: BMI, body mass index; CAP, controlled attenuation parameter; FAST, FibroScan-AST; FIB-4, Fibrosis-4; ALT, alanine aminotransferase; AST, aspartate aminotransferase; TBIL, total bilirubin; FBG, fasting blood glucose; TC, total cholesterol; LDL, low-density lipoprotein; HDL, high-density lipoprotein; TG, triglycerides; Hp, Helicobacter pylori; CAG, chronic atrophic gastritis; CAD, coronary artery disease; and GERD, gastroesophageal reflux disease.

**Table 2:** Characteristics according to the SIBO positivity in MASLD patients.

Variables	Negative (*n* = 825)	Positive (*n* = 1724)	*p*
Age	52.7 ± 13.8	54.0 ± 13.84	0.033
Body mass index	24.8 ± 3.2	25.3 ± 3.3	0.003
FAST score	0.13 (0.06, 0.25)	0.17 (0.07, 0.42)	<0.001
FIB-4 score	1.23 (0.93, 1.78)	1.34 (0.96, 1.96)	0.016
ALT	22.50 (15.40, 37.20)	24.70 (15.95, 40.25)	0.043
AST	23.00 (18.83, 30.10)	24.60 (19.00, 35.05)	0.021
TBIL	11.70 (9.12, 16.98)	12.40 (9.30, 16.88)	0.351
FBG	5.51 ± 1.60	5.55 ± 1.72	0.727
TC	4.71 ± 1.16	4.59 ± 1.12	0.076
LDL	2.84 ± 0.92	2.82 ± 1.13	0.768
HDL	1.15 ± 0.35	1.13 ± 0.33	0.32
TG	1.74 (1.24, 2.54)	1.69 (1.21, 2.35)	0.298
Male	525 (63.64)	1156 (67.05)	0.089
Smoking	277 (33.58)	569 (33.00)	0.775
Drinking	193 (23.39)	483 (28.02)	0.013
S			0.073
S1	194 (23.52)	341 (19.78)	
S2	258 (31.27)	590 (34.22)	
S3	373 (45.21)	793 (46.00)	
F			0.01
F0–F1	695 (84.24)	1385 (80.34)	
F2	57 (6.91)	112 (6.50)	
F3	43 (5.21)	115 (6.67)	
F4	30 (3.64)	112 (6.50)	
Hp	175 (23.74)	360 (23.05)	0.712
Gastric polyps	34 (4.12)	70 (4.06)	0.942
Intestinal polyps	92 (11.15)	257 (14.91)	0.01
CAG	54 (6.55)	131 (7.60)	0.338
CAD	35 (4.24)	135 (7.83)	<0.001
Hyperlipidemia	145 (17.58)	281 (16.30)	0.419
Hypertension	174 (21.09)	331 (19.20)	0.262
Diabetes	71 (8.61)	201 (11.66)	0.02
GERD	112 (13.58)	393 (22.80)	<0.001
Appendectomy	35 (4.24)	84 (4.87)	0.481
Cholecystectomy	67 (8.12)	115 (6.67)	0.183

Note: Continuous variables are now clearly labeled as either means ± SD for normally distributed data or medians (interquartile range) for non-normally distributed data. Categorical variables are presented as n (%). Abbreviations: BMI: Body Mass Index; FAST, Fatty Liver Severity Tool (a score to identify at-risk MASH); FIB-4, Fibrosis-4 Index (a non-invasive score to assess liver fibrosis); ALT, Alanine Aminotransferase; AST, Aspartate Aminotransferase; TBIL, total Bilirubin; FBG, fasting blood glucose; TC, total cholesterol; LDL, low-density lipoprotein; HDL, high-density lipoprotein; TG, Triglycerides; S, steatosis degree (S1, mild, S2, moderate, S3, severe); F, fibrosis stage (F0–F1, normal/mild, F2, moderate, F3, severe, F4, cirrhosis); Hp, Helicobacter pylori; CAG, chronic atrophic gastritis; CAD, coronary artery disease; Diabetes, diabetes mellitus; and GERD, gastroesophageal reflux disease.

## Data Availability

The datasets used in this study are available upon reasonable request to the corresponding author.

## Supplementary Materials

The following supporting information can be downloaded at: https://www.mdpi.com/article/10.3390/biomedicines14051042/s1. Figure S1. Distribution of SIBO subtypes of demographic characteristics. Stacked bar charts (left panels) and standardized residual heatmaps (right panels) displaying the proportion of SIBO stratified by (**A**) gender, (**B**) alcohol consumption, and (**C**) BMI categories. Standardized residuals > |2| indicate significant deviation from the expected frequencies. * *p* < 0.05, ** *p* < 0.01, and *** *p* < 0.001. Figure S2. Distribution of SIBO subtypes of comorbidity. (**A**) CAD, (**B**) GERD, and (**C**) intestinal polyps. Standardized residuals > |1.96| indicate significant deviation from expected frequencies. * *p* < 0.05, ** *p* < 0.01, and *** *p* < 0.001.
